# Mobile Genetic Elements Harboring Antibiotic Resistance Determinants in *Acinetobacter baumannii* Isolates From Bolivia

**DOI:** 10.3389/fmicb.2020.00919

**Published:** 2020-05-13

**Authors:** Mónica Cerezales, Kyriaki Xanthopoulou, Julia Wille, Oleg Krut, Harald Seifert, Lucía Gallego, Paul G. Higgins

**Affiliations:** ^1^Faculty of Medicine and Nursing, Department of Immunology, Microbiology, and Parasitology, University of the Basque Country UPV/EHU, Leioa, Spain; ^2^Institute for Medical Microbiology, Immunology and Hygiene, University of Cologne, Cologne, Germany; ^3^German Centre for Infection Research (DZIF), Partner Site Bonn-Cologne, Cologne, Germany; ^4^Paul-Ehrlich-Institute, Federal Institute for Vaccines and Biomedicine, Langen, Germany

**Keywords:** *A. baumannii*, plasmids, mobile genetic elements, antimicrobial resistance, carbapenemase

## Abstract

Using a combination of short- and long-read DNA sequencing, we have investigated the location of antibiotic resistance genes and characterized mobile genetic elements (MGEs) in three clinical multi-drug resistant *Acinetobacter baumannii*. The isolates, collected in Bolivia, clustered separately with three different international clonal lineages. We found a diverse array of transposons, plasmids and resistance islands related to different insertion sequence (IS) elements, which were located in both the chromosome and in plasmids, which conferred resistance to multiple antimicrobials, including carbapenems. Carbapenem resistance might be caused by a *Tn2008* carrying the *bla*_OXA–23_ gene. Some plasmids were shared between the isolates. Larger plasmids were less conserved than smaller ones and they shared some homologous regions, while others were more diverse, suggesting that these big plasmids are more plastic than the smaller ones. The genetic basis of antimicrobial resistance in Bolivia has not been deeply studied until now, and the mobilome of these *A. baumannii* isolates, combined with their multi-drug resistant phenotype, mirror the transfer and prevalence of MGEs contributing to the spread of antibiotic resistance worldwide and require special attention. These findings could be useful to understand the antimicrobial resistance genetics of *A. baumannii* in Bolivia and the difficulty in tackling these infections.

## Introduction

*Acinetobacter baumannii* is a non-fermenting Gram-negative bacilli and it is the second most common species after *Pseudomonas aeruginosa* in this group causing bacterial infections (Gonzalez-Villoria and Valverde-Garduno, 2016). While *A. baumannii* has been isolated from the wider environment such as water, soil, and animals, most studied isolates come from clinical samples, where *A. baumannii* has become a serious health problem, particularly in the intensive care unit, where it can cause serious and prolonged outbreaks (Gonzalez-Villoria and Valverde-Garduno, 2016). *A. baumannii* is often multidrug resistant (Peleg et al., 2008; Gonzalez-Villoria and Valverde-Garduno, 2016) making antimicrobial therapy of *A. baumannii* infections difficult. In some cases, with the advent of resistance to last line antibiotics such as colistin, there are few therapeutic options left (Higgins et al., 2010; Manchanda et al., 2010; Göttig et al., 2014; Cayô et al., 2016).

*Acinetobacter baumannii* is known to have a great genome plasticity, which is the capacity to acquire and disseminate genes, especially those related to antimicrobial resistance which are commonly associated with insertion sequence (IS) elements in transposons and plasmids; this dynamism in the genome of *A. baumannii* contributed to the rapid evolution of drug resistance (Adams et al., 2010) as has been demonstrated for IS*Aba1* mobilizing antimicrobial resistance genes (Mugnier et al., 2009). These processes are achieved thanks to mobile genetic elements (MGEs) harboring resistance genes. The simplest MGEs are ISs, that can also form transposons (Tn), and there are more complex structures such as integrons, resistance islands (RI), and plasmids. Antimicrobial resistance genes are often integrated into resistance cassettes related to translocation elements, causing cumulative resistance to multiple drugs (Roca et al., 2012).

A diverse range of MGEs have been described in *A. baumannii*, for example transposons such as *Tn2008, Tn2008B*, *Tn2006*, *Tn2009*, or *Tn2007*, which represent different transposon configurations carrying the *bla*_OXA–23_ gene together with IS*Aba1* or IS*Aba4*, and additional genes (Nigro and Hall, 2016). Great variability in antimicrobial resistance platforms, including MGEs, have been recorded even within the same international clone (IC), illustrating their contribution to the evolution of drug resistance (Adams et al., 2010). Plasmids in *Acinetobacter* spp. are unique and unrelated to those from other genera, although they often share the same resistance determinants, such as *strA, strB*, *tet(B)* or *sul2*. In *A. baumannii*, a diverse array of plasmids have been found, ranging in size from 2 Kb to more than 150 Kb. The larger plasmids normally encode for more than one resistance gene, but up to now little is known about these plasmids (Carattoli, 2013; Hamidian et al., 2016).

The aim of this study was to characterize the MGEs such as plasmids and RI of three different *A. baumannii* clinical isolates, representing different clonal lineages.

## Materials and Methods

### Bacterial Isolates

Three *A. baumannii* isolates recovered from two hospitals in Cochabamba, Bolivia, in September 2015, January 2016, and October 2016 (Table 1) representing three different ICs (IC4, IC5. and IC7) were selected for this study. We previously reported their carbapenem resistance mechanisms and molecular epidemiology (Cerezales et al., 2019).

**TABLE 1:** *Acinetobacterbaumannii* isolates data.

**Isolate**	**Molecularepidemiology**					**MIC (mg/L) for various antimicrobials**														
	**STs Ox/Pas**	**IC**	***bla*_OXA–51–like_**	**Sample origin**	**GenBank accession number**	**AMK**	AZI*	**CHL**	**CIP**	SXT*	**CST**	ERY*	**GEN**	**IPM**	KAN*	**LVX**	**MEM**	MIN*	TET*	TGC*
MC1	1518/991	IC7	*bla*_OXA–64_	Catheter	NZ_QXPV00 000000.1	>128 R	64	>128 R	>128 R	128	1 S	64	32 R	32 R	>256	32 R	64 R	64	>128	16
MC23	1520/79	IC5	*bla*_OXA–65_	Urine	NZ_QXPJ00 000000.1	>128 R	64	>128 R	128 R	128	1 S	64	>128 R	1 S	>256	32 R	2 S	1	16	4
MC75	236/15	IC4	*bla*_OXA–51_	Ulcer	NZ_QXOL000 00000.1	>128 R	32	128 R	>128 R	128	1 S	32	>128 R	32 R	>256	8 R	64 R	0,5	16	2

AMK, amikacin; AZI, azithromycin; CHL, chloramphenicol; CIP, ciprofloxacin; SXT, trimethoprim-sulfamethoxazole; CST, colistin; ERY, erythromycin; GEN, gentamicin; IPM, imipenem; KAN, kanamycin; LVX, levofloxacin; MEM, meropenem; MIN, minocycline; TET, tetracycline; TGC, tigecycline. * No EUCAST breakpoints are available for these antimicrobials.

### Antimicrobial Susceptibility Testing

In addition to previously reported carbapenem susceptibility testing results, in the present study we investigated the following antimicrobials by agar dilution: amikacin, azithromycin, chloramphenicol, trimethoprim-sulfamethoxazole, erythro- mycin, levofloxacin, minocycline, kanamycin, and tetracycline. MICs were interpreted using the European Committee on Antimicrobial Susceptibility Testing (EUCAST) breakpoints^1^.

### MinION Long-Read Sequencing and Assembly

The Oxford Nanopore Technologies (Oxford, United Kingdom) MinION sequencer was used to obtain long reads to span repetitive elements and close genomes and plasmids. DNA extraction was performed using the Genomic-tip 100/G kit (Qiagen, Hilden, Germany). Library preparation was carried out according to manufacturer’s indications using a combination of Native Barcoding Kit 1D and Ligation Sequencing Kit 1D; EXP-NBD103 and SQK-LSK108 (Oxford Nanopore Technologies, Oxford, United Kingdom), respectively.

The tool Albacore (Oxford Nanopore Technologies, Oxford, United Kingdom) was used for demultiplexing the reads which were later used to perform the Canu assembly (Koren et al., 2017). A hybrid assembly combining previous MiSeq short reads with MinION-generated long reads was performed using a hybridSpades (Antipov et al., 2016).

### Plasmid Annotation and Visualization

ORFfinder (NCBI)^2^ was used to predict the open reading frames (ORF) of the plasmids. A second functional annotation of the genomes was performed using the online tool Rapid Annotation Subsystem Technology (RAST)^3^ (Genomics et al., 2008). Subsequently, the tool SnapGene Viewer (GSL Biotech)^4^ was used to obtain a circular diagram of the plasmids. Graphic comparisons between similar plasmids, pMC1.1 and pA297-3, as well as pMC23.1 and pAC30c, were carried out with the tool Kablammo (Wintersinger and Wasmuth, 2015).

### Conjugation Experiments

Broth mate conjugation experiments were performed to determine the location of antimicrobial resistance genes using the sodium azide-resistant *Escherichia coli* J53 and the rifampicin-resistant *A. baumannii* BM4547 as recipient strains. Selection of *E. coli* J53 transconjugants was performed using sodium azide (200 mg/L) combined either with amikacin (30 mg/L), streptomycin (30 mg/L), kanamycin (30 mg/L), gentamicin (30 mg/L), or ticarcillin (100 mg/L), and selection for *A. baumannii* BM4547 was performed using rifampicin (60 mg/L) combined with gentamicin (30 mg/L) or ticarcillin (100 mg/L. Transconjugants were selected with the antimicrobials to select for the plasmids encoding their respective resistance genes. Strain MC1 was resistant to rifampicin, therefore conjugation with *A. baumannii* BM4547 could not be performed. The transconjugants were tested by PCR for the *bla*_TEM_ gene.

## Results and Discussion

MC1 and MC75 were previously tested as carbapenem-resistant and carried the carbapenemase encoding *bla*_OXA–23–like_ gene (Cerezales et al., 2019). Further testing revealed that MC1 and MC75 were also resistant to amikacin, chloramphenicol, ciprofloxacin, gentamicin, and levofloxacin. MC23 was resistant to amikacin, chloramphenicol, ciprofloxacin, gentamicin, and levofloxacin but was susceptible to carbapenems. All three isolates were susceptible to colistin (Table 1).

The *bla*_OXA–23_ encoding gene was located on the chromosome in a *Tn2008* vehicle in the isolates MC1 and MC75 (Table 2). In *A. baumannii*, the *bla*_OXA–23–like_ gene is associated with IS*Aba1*, which contributes to its overexpression as well as its mobilization (Nigro and Hall, 2016). *Tn2008* has previously been described in Bolivian *A. baumannii* isolates and this mirrors the spread of this structure among different ICs leading to a carbapenem-resistant phenotype (Nigro and Hall, 2016; Sennati et al., 2016; Chen et al., 2017; Ewers et al., 2017; Cerezales et al., 2018).

**TABLE 2:** Plasmid content, size, location resistance genes as determined by WGS, and accession numbers.

				**Location of resistance genes**	
**Isolate**	**Plasmids**	**Accession number**	**Size**	**Plasmid**	**Chromosome**
MC1	pMC1.1	MK531536	184 Kb	*strA* *strB* *aac(3)-IIa* *aac(6′)-Ian* *tet(B)* *sul2*	*bla*_OXA–23_
	pMC1.2	MK531537	8.7 Kb		
MC23	pMC23.1 pMC23.2 pMC23.3	MK531538 MK531537 MK531539	67 Kb 8.7 Kb 6 Kb	*aadB*	*strA* *strB* *sul2* *floR* *aadA1* *sat2* *dfrA1*
MC75	pMC75.1	MK531540	149 Kb	*strA* *strB* *sul2*	*bla*_OXA–23_ *aphA6*
	pMC75.2	MK531541	13.9 Kb	*bla*_TEM–1_	
				*aac(3)-IIa*	

### Resistance Islands

**FIGURE 1:**
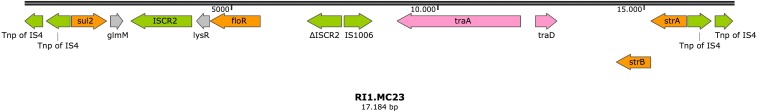
Resistance island RI1.MC23 in isolate MC23. Arrows represent predicted ORFs and the direction of the arrow represents the direction of transcription. Resistance genes are shown by orange arrows and transposon-related genes, recombinases, and insertion sequences are indicated by green arrows. Genes involved in plasmid mobility are shown in pink. Other genes are indicated by gray arrows. Hypothetical proteins are not shown.

In the isolate MC23, the gene *strA* was located on a resistance island in the chromosome (RI1.MC23) (accession number MK531542), together with other antimicrobial resistance genes such as *sul2, floR*, and *strB*. Diverse IS elements were found, with the resistance island bracketed by two copies of a transposase from the IS4 family in reverse orientation (Figure 1). Two genes involved in conjugation were also present in this structure, suggesting a plasmid origin.

**FIGURE 2:**
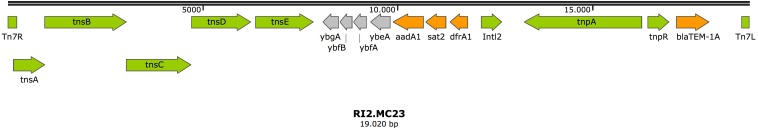
Resistance island RI2.MC23 in isolate MC23. Arrows represent predicted ORFs and the direction of the arrow represents the direction of transcription. Resistance genes are shown by orange arrows and transposon-related genes, recombinases, and insertion sequences are indicated by green arrows. Other genes are indicated by gray arrows. Hypothetical proteins are not shown.

In addition, a second chromosomal resistance island was also found in this isolate (RI2.MC23) (accession number MK531543), that carried a typical structure from class 2 integrons, *dfrA-sat2-aadA1-ybeA-ybfAybfB-ybgA*, located between the Tn7 transposition module *tns*ABCDE and a non-functional Intl2 integrase. Additionally, a Tn3 transposon was found inserted in the Tn7 transposon, carrying three genes, *tnpA*, encoding for a Tn3 transposase; *tnpR* encoding a Tn3 resolvase; and the antimicrobial resistance gene *bla*_TEM–1A_ (Figure 2).

The gene encoding Apha6 was found on the chromosome of MC75 bracketed by two IS*Aba125* that is a composite transposon known as Tn*aphA6* (Matos et al., 2019).

### Plasmids

#### pMC1.1

**FIGURE 3:**
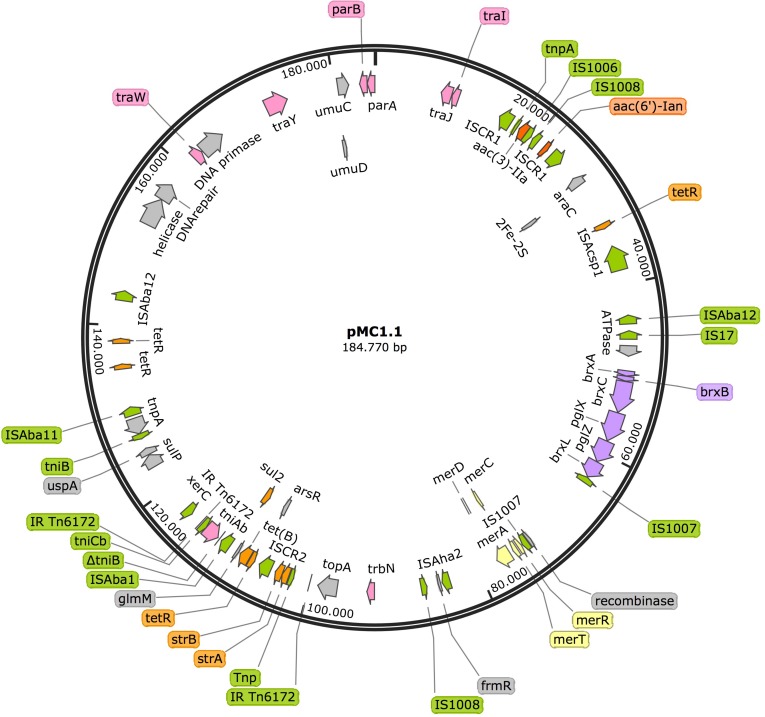
Plasmid pMC1.1 in isolate MC1. Arrows represent predicted ORFs and the direction of the arrow represents the direction of transcription. Resistance genes are shown by orange arrows and transposon-related genes, recombinases and insertion sequences are indicated by green arrows. Transfer protein encoding genes, conjugal transfer protein encoding genes, and genes involved in plasmid partition and replication are shown in pink. The mercury resistance operon genes are indicated by yellow arrows and the BREX type 1 system is shown in purple. Other genes are indicated by gray arrows. Hypothetical proteins are not shown.

**FIGURE 4:**
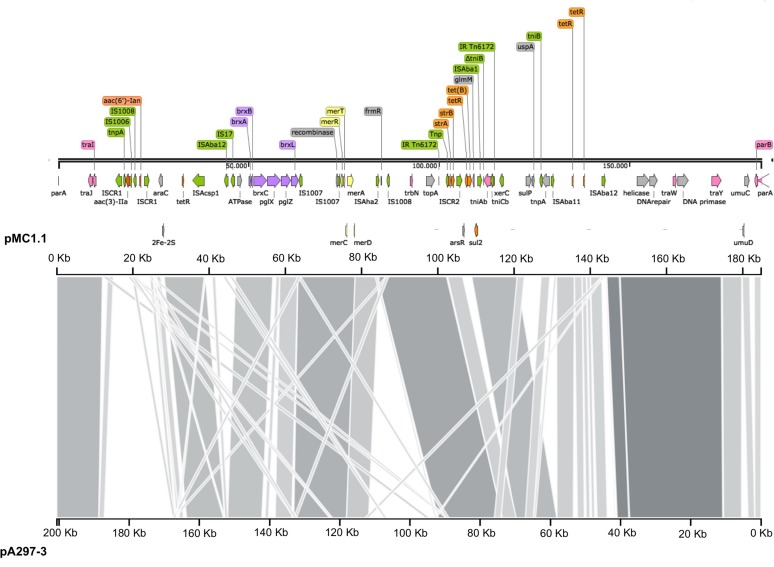
Comparison of plasmids pMC1.1 and pA297-3. The top axis represents the pMC1.1, the bottom axis represents pA297-3. Gray shaded regions show the homologous regions between the two plasmids.

Annotation of pMC1.1 (accession number MK531536), 39% GC content, revealed many different IS elements such as IS*1006*, IS*1007*, IS*1008*, IS*Acsp1*, IS91 family, IS*Aha2*, IS*Aba11*, IS*Aba12*, and IS*17*. This plasmid carried a mercuric resistance operon, similar to an already described mercuric Tn in a 200 Kb plasmid (pA297-3) from an IC1 *A. baumannii* isolate, but it lacks the *merP* open reading frame (Hamidian et al., 2016). Different antimicrobial resistance determinants such as *strA, strB*, *aac(3)-IIa*, and *aac(6′)-Ian*, conferring resistance to aminoglycosides, *sul2* conferring resistance to sulphonamides, and *tet(B)* conferring resistance to tetracycline were also present. The region of the plasmid carrying *strA, strB, and sul2* shared high homology with *Tn6172*, located in pA297-3 as well (Figure 3), however, in pMC1.1 *arsR, tetR*, and *tet(B)* genes were also located within *Tn6172* with an IS*CR2* transposable element (IS91 family). This IS*CR* element has been described associated with different antimicrobial resistance genes in *A. baumannii*, especially with *sul2*, contributing to their mobilization thanks to a rolling circle transposition mechanism (Toleman et al., 2006), and was similar to other plasmids from Argentina (Vilacoba et al., 2013) and to plasmids found in an ST25 isolate from Australia (Hamidian and Hall, 2016). However, the location of *tetR-tetB* genes was different; they were located between *glmM* and *arsR*, suggesting a possible later insertion of these genes in different positions within the transposon (Vilacoba et al., 2013). In addition, the same inverted repeats (IR) generated by the insertion of the transposon were also found in pMC1.1 which together with the similar backbone with pA297-3 (Figure 4) suggest they share a common origin. The genes *aac(3)-IIa* and *aac(6′)-Ian* were associated with IS6 family IS and bracketed by two IS*CR1* in inverted orientation. IS*CR1* belongs to the IS91 family and has been described related to class 1 integrons and antimicrobial resistance genes in diverse Gram-negative species such as *Klebsiella pneumoniae*, *P. aeruginosa*, and *Citrobacter freundii* (Toleman et al., 2006). Different transfer genes (*tra*) were also found in this plasmid, as well as genes involved in plasmid partition and replication (*parB/repB* and *xerC*) that are related to segregational stability of plasmids. This plasmid also encoded a system called BREX type 1 (bacteriophage exclusion) which has been described to be involved in phage resistance (Goldfarb et al., 2015).

#### pMC1.2/pMC23.2

**FIGURE 5:**
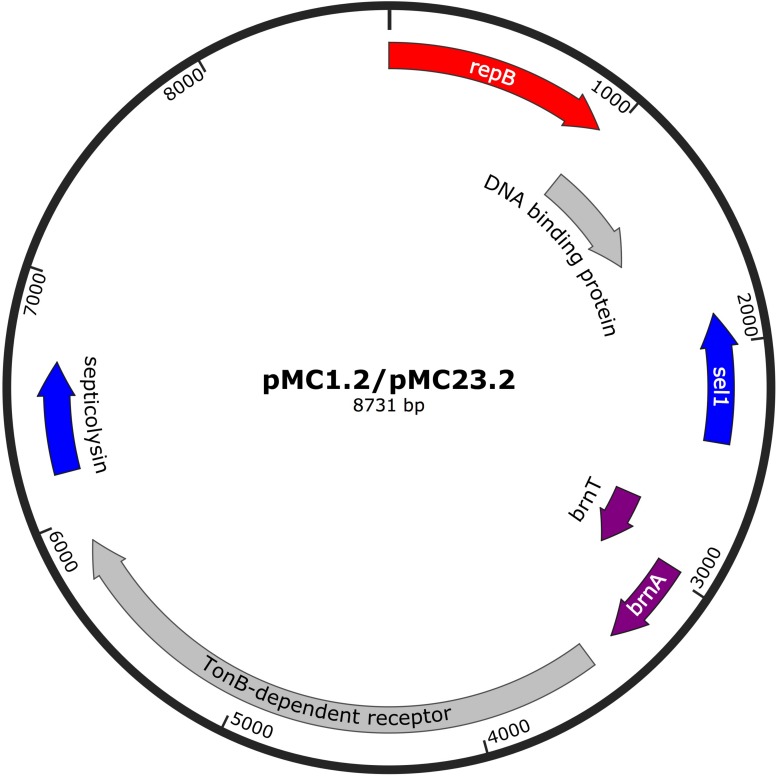
Plasmids pMC1.2 and pMC23.2 in isolates MC1 and MC23, respectively. Arrows represent predicted ORFs and the direction of the arrow represents the direction of transcription. Red arrow is used for the replicon. The toxin-antitoxin system is indicated by violet arrows and blue arrows represent virulence genes. Other genes are shown in gray.

The 8.7 Kb plasmids found in MC1 and MC23 (pMC1.2 and pMC23.2) were identical (accession number MK531537), with a GC content of 34.3% (Figure 5). This small plasmid has often been found in IC1 *A. baumannii* isolates (Lean and Yeo, 2017). Annotation of this plasmid revealed ORFs encoding for a RepB replicon (Rep-3 superfamily, GR2) (Bertini et al., 2010; Lean and Yeo, 2017) a toxin-antitoxin system (BrnT-BrnA), that is involved in vertical stability; TonB-dependent receptor, related to the transmission of signals from the outside of the cell leading to transcriptional activation of target genes; a *septicolysin* gene encoding a cytolytic enzyme toward eukaryotic cells and is involved in pathogenesis; as well as *sel1* gene that encodes for a protein that has been described in diverse prokaryotic genera and has an important role in virulence.

#### pMC23.1

**FIGURE 6:**
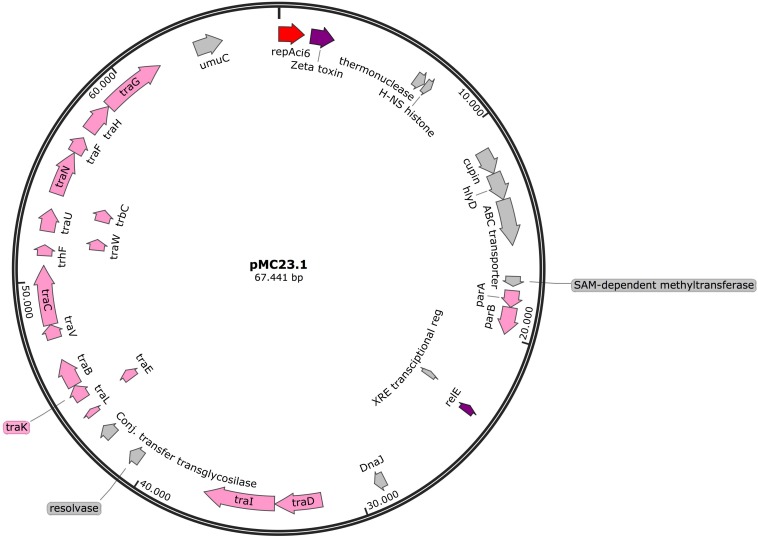
Plasmid pMC23.1 in isolate MC23. Arrows represent predicted ORFs and the direction of the arrow represents the direction of transcription. Red arrow is used for the replicon. The toxins are indicated by violet arrows represent virulence genes. Transfer protein encoding genes, conjugal transfer protein encoding genes, and genes involved in plasmid partition and replication are shown in pink. Other genes are shown in gray. Hypothetical proteins are not shown.

**FIGURE 7:**
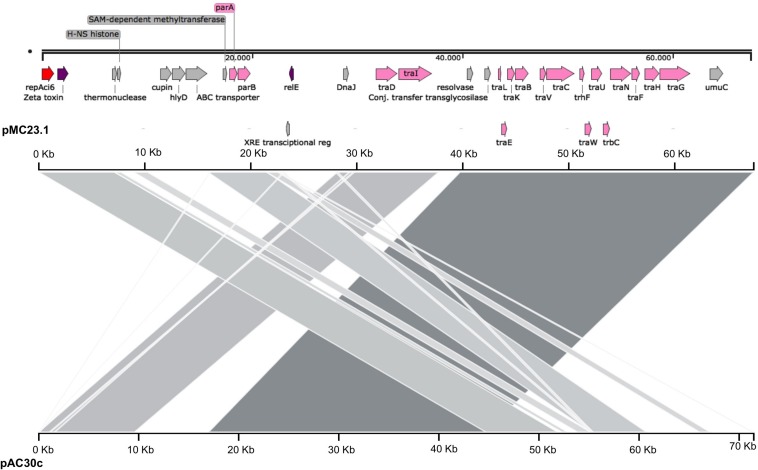
Comparison of plasmids pMC23.1 and pAC30c. The top axis represents the pMC23.1, the bottom axis represents pAC30c. Gray shaded regions show the homologous regions between the two plasmids.

The largest plasmid in MC23 was the 67.5 Kb pMC23.1 (accession number MK531538) (Figure 6). It belonged to GR6 according to its replicase, *repAci6*. Its GC content was 33.7% and almost all of its putative protein encoding genes were related to conjugative plasmid transfer in a *tra* locus, some of them are part of a type IV (T4SS) secretion system. This T4SS is able to secrete or take up both proteins and DNA, and possibly is involved in natural competence, a feature of *A. baumannii* (Salto et al., 2018). Two toxin encoding genes were present in the plasmid, *relE* and zeta toxin, but no antitoxins were found, although they were present in a very similar plasmid (pAC30c) in an *A. baumannii* isolate belonging to ST195 (IC2) (Figure 7; Lean et al., 2016). In addition, the partition genes *parA/parB* were also encoded on pMC23.1. The backbone of pMC23.1 and pAC30c were very similar, with only a few differences. pMC23.1 lacked some hypothetical proteins present in pAC30c, and the region encoding for tellurite resistance (*telA* gene and IS*66*); while *traD*, a cupin-like protein (that is a superfamily of enzymes including dioxygenases, decarboxylases, hydrolases, or isomerases); HlyD protein, that exports proteins from the cytosol to the outside of the cell, and an ABC transporter were not present in pAC30.

#### pMC23.3

A 6 Kb small plasmid was present in the isolate MC23, pMC23.3 (accession number MK531539), 39.2% GC content, and was found to have 100% similarity with an already described plasmid, pRAY from an isolate in South Africa, encoding resistance to gentamicin, kanamycin and tobramycin (*aadB* gene) together with *mobA* and *mobC* genes, which are thought to encode mobilization proteins (Lean and Yeo, 2017). Many similar plasmids have been found in diverse *A. baumannii* isolates from different ICs and countries, suggesting a common origin and subsequent diversification in their evolution. Concurrent with other studies, no *rep* gene was found in the plasmid sequence, supporting the idea of the presence of a mechanism of replication relying in the host RNA polymerase (Lean and Yeo, 2017).

#### pMC75.1

**FIGURE 8:**
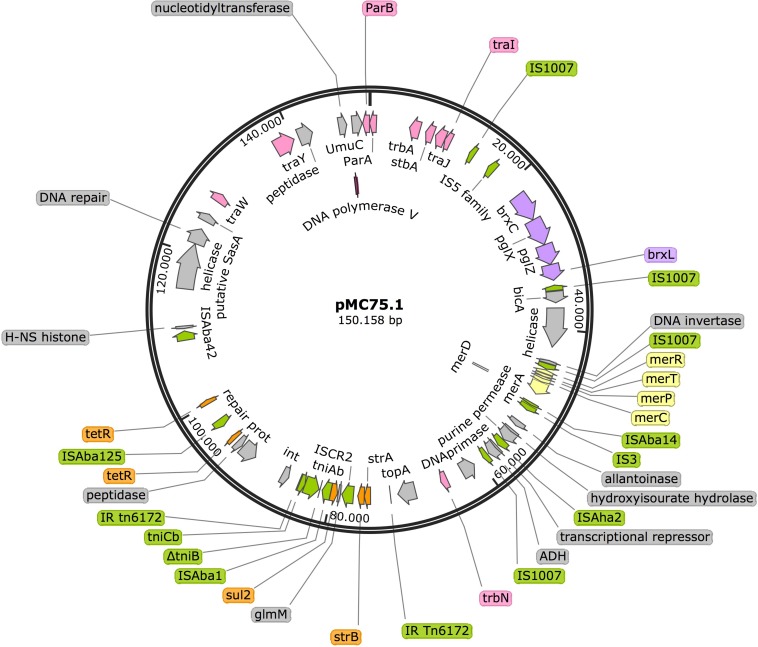
Plasmid pMC75.1 in isolate MC75. Arrows represent predicted ORFs and the direction of the arrow represents the direction of transcription. Resistance genes are shown by orange arrows and transposon-related genes, recombinases and insertion sequences are indicated by green arrows. Transfer protein encoding genes, conjugal transfer protein encoding genes, and genes involved in plasmid partition and replication are shown in pink. The mercury resistance operon genes are indicated by yellow arrows and the BREX type 1 system is shown in purple. Other genes are indicated by gray arrows. Hypothetical proteins are not shown.

Analysis of pMC75.1 (accession number MK531540) a large plasmid of 150 Kb revealed that it was very similar to pMC1.1 (sharing 80% of their sequences), it also carried a Tn*6172*, in which antimicrobial resistance genes such as *sul2, strB*, and *strA* are encoded, but lacking *tet(B)* and *arsR* that were present in pMC1.1 (Figure 8). The *mer* operon was also found in this plasmid, and many genes encoding conjugative transfer proteins. The BREX type 1 system was also present. A *stbA* gene was found, the protein encoded by this gene plays a role in plasmid stability as well as *parA/parB*. Several IS elements were also present, i.e., IS*Aba1*, IS*Aba125*, IS*Aba14*, IS*Aba42*, IS*1007*, and IS*Aha2*. However, this plasmid lacked the transposon carrying *aac(3)-IIa* and *aac(6′)-Ian*.

#### pMC75.2

**FIGURE 9:**
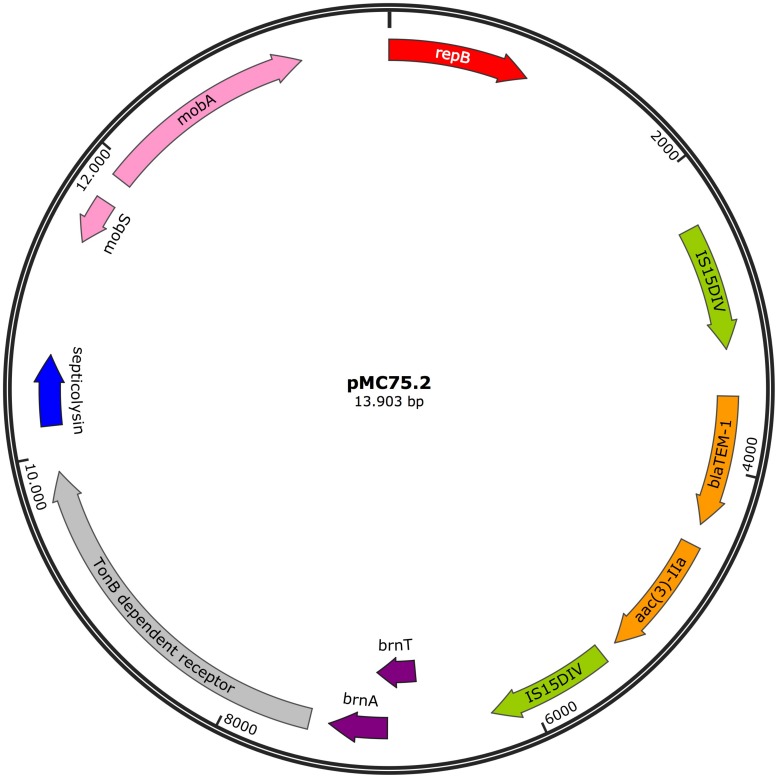
Plasmid pMC75.2 in isolate MC75. Arrows represent predicted ORFs and the direction of the arrow represents the direction of transcription. Resistance genes are shown by orange arrows and insertion sequences are indicated by green arrows. Genes involved in plasmid mobility are shown in pink. The toxin-antitoxin system is shown in violet. Blue represents virulence genes. Other genes are indicated by gray arrows. Hypothetical proteins are not shown. Red arrow is used for the replicon.

The 13.9 Kb plasmid, pMC75.2 (accession number MK531541) (Figure 9) with a GC content of 40.3%, carried the broad-spectrum β-lactamase *bla*_TEM–1B_ and the aminoglycoside resistance gene *aac(3)-IIa* flanked on both sides by IS15DIV; a toxin-antitoxin system, *brnT/brnA*; a TonB-dependant receptor, a septicolysin gene and *mobA/mobS*, which are involved in plasmid mobility. Conjugation experiments revealed that pMC75.2 was transferable into *A. baumannii* BM4547 but it was unstable and was lost after several passages. The replicon of this plasmid belonged to the RepB (Rep_3) superfamily with 100% homology. This plasmid shares a great homology with pMC1.2/pMC23.2, same RepB, toxin-antitoxin system, TonB-dependant receptor and septicolysin; it seems that one of them has lost or alternatively acquired the integron carrying the antimicrobial resistance genes and the mobility genes.

Recently, two similar plasmids to pMC75.1 and pMC75.2 were described in a Brazilian *A. baumannii* isolate representing the same ST (ST15). This illustrates that these plasmids can be very plastic by acquiring or losing genes, but can also be conserved within a ST (Matos et al., 2019).

The two carbapenem-resistant isolates carried the *bla*_OXA–23_ gene in Tn*2008*, which has been previously described in diverse ICs (Nigro and Hall, 2016; Ewers et al., 2017) including IC7 isolates recovered from a hospital in the same city, Cochabamba (Sennati et al., 2016). The Tn*2008* contributes to the overexpression of the carbapenemase encoding gene and to its mobilization. In addition, all three isolates harbored three aminoglycoside resistance genes such as *aac(3)-IIa*, *strA*, and *strB*; and *sul2* conferring resistance to sulphonamides; MC1 carried *tetB* conferring resistance to tetracycline as well. All the genes were found to be associated with IS elements, constituting transposons that lead to their mobilization and make genetic rearrangements more likely to happen. These genes were found both in the chromosome and in plasmids, demonstrating the plasticity of the *A. baumannii* genome and the mobility of these antimicrobial resistance determinants within MGEs such as transposons or plasmids.

## Conclusion

In summary, these data further confirm that *A. baumannii* has a great ability to acquire antimicrobial resistance determinants and become a threat in hospitals. These are associated with different plasmids and many different IS elements, of which some are found in multiple genera. For these reasons it is important to study the dynamics and resistomes of the bacterial populations in order to understand the situation in each hospital or unit. The fact that some of these plasmids have been found in diverse *A. baumannii* clonal lineages mirrors the transfer and prevalence of these MGEs contributing to the spread of antimicrobial resistance worldwide.

## Data Availability Statement

The datasets generated for this study can be found in the GenBank, MK531536, MK531538, MK531537, MK531539, MK531540, and MK531541.

## Author Contributions

MC, KX, JW, and PH contributed to the design of the experiments. MC, KX, and JW performed the experiments. MC, KX, JW, OK, HS, LG, and PH analyzed and interpreted the data. MC, KX, and PH wrote the manuscript. All authors contributed to critical manuscript revision, read, and approved the submitted version.

## Conflict of Interest

The authors declare that the research was conducted in the absence of any commercial or financial relationships that could be construed as a potential conflict of interest.

## Footnotes

1 http://www.eucast.org/clinical_breakpoints/

2 https://www.ncbi.nlm.nih.gov/orffinder/

3 https://rast.nmpdr.org/

4 https://www.snapgene.com
